# Clinical Characteristics and Diagnostic Prediction of Severe Fever with Thrombocytopenia Syndrome and Rickettsiosis in the Co-Endemic Wakayama Prefecture, Japan

**DOI:** 10.3390/medicina59112024

**Published:** 2023-11-17

**Authors:** Kan Teramoto, Shinobu Tamura, Kikuaki Yoshida, Yukari Inada, Yusuke Yamashita, Masaya Morimoto, Toshiki Mushino, Daisuke Koreeda, Kyohei Miyamoto, Nobuhiro Komiya, Yoshio Nakano, Yusaku Takagaki, Yusuke Koizumi

**Affiliations:** 1 Department of Internal Medicine, National Health Insurance Susami Hospital, Wakayama 649-2621, Japan; 2 Department of Hematology, Kinan Hospital, Wakayama 641-8509, Japan; 3 Department of Hematology/Oncology, Wakayama Medical University, Wakayama 641-8509, Japan; 4 Department of Infection Control and Prevention, Wakayama Medical University, Wakayama 641-8509, Japan; 5 Department of Emergency and Critical Care Medicine, Wakayama Medical University, Wakayama 641-8509, Japan; 6 Department of Emergency and Intensive Care Medicine, Japanese Red Cross Wakayama Medical Center, Wakayama 640-8558, Japan; 7 Department of Infectious Diseases, Japanese Red Cross Wakayama Medical Center, Wakayama 640-8558, Japan; 8 Department of Internal Medicine, Kinan Hospital, Wakayama 646-8588, Japan

**Keywords:** tick-borne infections, severe fever with thrombocytopenia syndrome, rickettsiosis, Japanese spotted fever, scrub typhus

## Abstract

*Background and Objectives*: The Wakayama prefecture is endemic for two types of tick-borne rickettsioses: Japanese spotted fever (JFS) and scrub typhus (ST). Severe fever with thrombocytopenia syndrome (SFTS) is a tick-borne hemorrhagic viral disease with a high mortality rate and is often difficult to differentiate from such rickettsioses. SFTS cases have recently increased in Wakayama prefecture. For early diagnosis, this study aimed to evaluate the clinical characterization of such tick-borne infections in the co-endemic area. *Materials and Methods*: The study included 64 febrile patients diagnosed with tick-borne infection in Wakayama prefecture between January 2013 and May 2022. Medical records of 19 patients with SFTS and 45 with rickettsiosis (JSF, *n* = 26; ST, *n* = 19) were retrospectively examined. The receiver operating curve (ROC) and area under the curve (AUC) were calculated to evaluate potential factors for differentiating SFTS from rickettsiosis. *Results*: Adults aged ≥70 years were most vulnerable to tick-borne infections (median, 75.5 years; interquartile range, 68.5–84 years). SFTS and rickettsiosis occurred mostly between summer and autumn. However, no significant between-group differences were found in age, sex, and comorbidities; 17 (89%) patients with SFTS, but none of those with rickettsiosis, experienced gastrointestinal symptoms such as vomiting, abdominal pain, and diarrhea. Meanwhile, 43 (96%) patients with rickettsiosis, but none of those with SFTS, developed a skin rash. The AUCs of white blood cells (0.97) and C-reactive protein (CRP) levels (0.98) were very high. Furthermore, the differential diagnosis of SFTS was significantly associated with the presence of gastrointestinal symptoms (AUC 0.95), the absence of a skin rash (AUC 0.98), leukopenia <3.7 × 10^9^/L (AUC 0.95), and low CRP levels < 1.66 mg/dL (AUC 0.98) (*p* < 0.001 for each factor). *Conclusions*: Clinical characteristics and standard laboratory parameters can verify the early diagnosis of SFTS in areas where tick-borne infections are endemic.

## 1. Introduction

Tick-borne infections are caused by the bite of pathogen-carrying ticks, and reported cases have recently been increasing in Japan, particularly in the western area [1,2,3,4,5,6]. Japanese spotted fever (JSF) and scrub typhus (ST) are representatives of rickettsiosis in Japan. JSF is a zoonosis transmitted by ticks carrying the pathogen *Rickettsia japonica*. ST is a tick-borne febrile disease caused by infection with *Orientia tsutsugamushi*. The distribution of these rickettsioses has expanded to the central and eastern areas [1,2,3]. The classic triad of rickettsiosis symptoms is a high fever, skin rash, and eschar. Moreover, these patients exhibit thrombocytopenia, elevated transaminases, and inflammatory markers. Tetracyclines are effective antibiotics against most rickettsioses [7]. A few patients with these rickettsioses become severely ill; however, the mortality rate of rickettsiosis, including JSF (0.91%) and ST (0.48%), is relatively low [2].

Severe fever with thrombocytopenia syndrome (SFTS) is an emerging viral infection caused by the tick-borne *Dabie bandavirus* (formerly called the SFTS virus) in the *Phenuiviridae* family [4,5,6]. In 2011, it was first identified in central China [8]. Since then, epidemics have occurred in several East Asian countries, including Japan. A female patient with SFTS was identified in the Yamaguchi prefecture in Japan [9]. Over time, SFTS cases have increased, mainly in western Japan, and continued to expand in the eastern region, and 50–60 cases are reported annually [10]. The major clinical manifestations of human SFTS include a high fever, thrombocytopenia, leukocytopenia, and gastrointestinal symptoms. However, SFTS is sometimes difficult to differentiate from rickettsioses because it presents with similar clinical findings (e.g., a high fever, eschar, thrombocytopenia, and hepatic dysfunction). Some SFTS cases rapidly deteriorate into multiple organ failure and require intensive care unit (ICU) admission [11,12,13].

Despite this rapid and lethal disease course, no antiviral treatment has proven to be effective against SFTS so far, except for rickettsioses [5,6]. Therefore, supportive treatment, such as blood transfusion, renal replacement therapy, and empirical antibiotics, has been a fundamental intervention for SFTS [5,6]. Globally, the mortality rate of SFTS ranges from 15.1% to 50% depending on hospital admission time, viral load, age, comorbidities, and abnormal laboratory findings [14,15]. Moreover, some reports have shown that there is person-to-person transmission of SFTS [16,17,18,19]. Hence, early diagnosis and prompt treatment of SFTS are crucial to improve the prognosis of such patients and prevent person-to-person transmission.

Patients with rickettsiosis and SFTS present similar epidemiological, geographical, and clinical manifestations; however, treatment and infection control strategies differ between the two infections [1,2,3,4,5,6,10,20,21]. Although these culprit pathogens can be confirmed by molecular and serological analyses, these laboratory tests generally require more time and special resources [5,6,22,23]. Thus, clinical differentiation between rickettsiosis and SFTS is important in planning the management of treatment and infection control during the initial presentation, particularly in the primary care setting. In Wakayama prefecture, we first reported a female patient with a mild clinical course of SFTS in 2014 [24]. Currently, Wakayama prefecture has become one of the hotspots of SFTS in addition to two rickettsioses. Specifically, three tick-borne infections often occur in the community of this prefecture. Therefore, simple methods are required to differentiate SFTS from rickettsiosis. This study aimed to clarify the differences between rickettsioses and SFTS based on common clinical presentations in the co-endemic area.

## 2. Materials and Methods

### 2.1. Patient and Study Design

In this study, febrile patients with newly diagnosed SFTS, JSF, and ST between January 2013 and May 2022 were included across four hospitals in Wakayama prefecture (namely, Wakayama Medical University Hospital, Japanese Red Cross Wakayama Medical Center, Kinan Hospital, and National Health Insurance Susami Hospital). In Wakayama Medical University Hospital, Japanese Red Cross Wakayama Medical Center, and Kinan Hospital, intensive care was provided for patients with severe illnesses. A definitive diagnosis was made by polymerase chain reaction (PCR) of blood or eschar samples, except in three cases (ST), which were diagnosed by paired serology. PCR was performed at the public health center of Wakayama [25], taking a few days for the results. Since the clinical characteristics of JSF and ST are clinically similar in Japan [20], they are collectively referred to as rickettsiosis in this study. The included patients were divided into two groups according to the diagnosis: patients with SFTS or those with rickettsiosis (ST and JSF). In this analysis, no patients had co-infection with SFTS and rickettsiosis. The medical records of these patients were then retrospectively analyzed.

This study was conducted in accordance with the Declaration of Helsinki and was approved by the Ethical Review Board of Wakayama Medical University (approval no. 3389; approval date: 25 January 2022). The opt-out method was applied to obtain informed consent for this study.

### 2.2. Clinical Data Collection

The following data were obtained retrospectively from patient medical records: sex, age, onset season, various symptoms, and laboratory tests. Comorbidities included hypertension, diabetes mellitus, hepatic disease, chronic kidney disease, pulmonary disease, heart disease, and cancer. Clinical manifestations included eschar, skin rash, and gastrointestinal symptoms such as vomiting, abdominal pain, and diarrhea. In all hospitals, common laboratory tests, namely, complete blood count (white blood cell (WBC), hemoglobin (Hb), and platelet (PLT)) and biochemical parameters (aspartate aminotransferase (AST), creatinine (Cr), lactate dehydrogenase (LDH), total bilirubin (T-Bil), creatine kinase (CK), and C-reactive protein (CRP)), were performed on admission. Moreover, the coagulation system (prothrombin time-international normalized ratio (PT-INR)) was also examined in all hospitals except for the National Health Insurance Susami Hospital.

### 2.3. Statistical Analyses

All statistical analyses were performed using Stata version 17.0 (StataCorp, College Station, TX, USA). Continuous variables were reported as medians and interquartile range (IQR). Categorical variables were presented as numbers with percentages. Differences between continuous variables were evaluated using the Wilcoxon rank-sum test, and differences between the proportions of categorical variables were assessed using the chi-squared test or Fisher’s exact test. Receiver operating characteristic (ROC) analyses were performed, and curves were generated to determine the best cut-off for differentiation between SFTS and rickettsiosis. The area under the ROC curve (AUC) was calculated as a measure of diagnostic effectiveness and classified as good (0.8–0.89) or excellent (0.9–1). To define the optimal cutoff point for the laboratory tests, Youden’s J-statistics were determined. For all statistical analyses, a *p*-value of < 0.05 was considered significant.

## 3. Results

### 3.1. Characteristics, Treatment, and Outcomes of Patients Diagnosed with Tick-Borne Infections

In total, 64 patients with tick-borne infections were included in this analysis. Among these tick-borne infections, 19 (29.7%) patients were finally diagnosed with SFTS and 45 (70.3%) were diagnosed with rickettsiosis. In the rickettsiosis group, 26 and 19 patients had JSF and ST, respectively. Table S1 compares the clinical characteristics between JSF and ST. The clinical findings more frequently observed in patients with ST were presentation over autumn–winter and the presence of eschar, consistent with a previous report [20]. The baseline characteristics of the patients at the time of the initial diagnosis of SFTS and rickettsiosis are shown in Table 1. The median ages of the patients with total tick-borne infections, SFTS, and rickettsiosis were 75.5 (IQR 68.5–84), 79 (IQR 69–85), and 74 (IQR 63–81) years, respectively. Nearly the same number of male and female patients had these infections. More than half of these patients had comorbidities such as hypertension and diabetes mellitus. These two groups were not significantly different in age, sex, or comorbidities. In Wakayama prefecture, SFTS and rickettsiosis occurs throughout the year. Most patients with SFTS (14/19 patients, 74%) and JSF (21/26 patients, 81%) were diagnosed in spring–summer (from March to August), whereas all ST cases occurred in autumn–winter (September–February). Significant differences were found in the season group (spring–summer and autumn–winter) between the SFTS and rickettsiosis groups (*p* = 0.047). Eschar was identified in 33 (73%) patients of the rickettsiosis group and in 9 (47%) in the SFTS group (*p* = 0.046). Among the clinical features, 17 (89%) patients with SFTS complained of gastrointestinal symptoms (vomiting, abdominal pain, and diarrhea); however, they did not all exhibit skin rashes. Conversely, 43 (96%) patients with rickettsiosis exhibited skin rashes, and none had gastrointestinal symptoms. Significant differences were noted in gastrointestinal symptoms and skin rashes between the two groups (*p* < 0.001).

All patients with rickettsiosis were treated with tetracyclines (Table 1). Nearly all of these patients showed improvements in their general condition and in the skin rashes. Among them, only one (2%) patient was transferred to the ICU; however, she died of multiorgan failure. The SFTS group was mainly treated with fluids and antimicrobials; some were treated with steroids and immunoglobulin replacement. Eight patients (42%) with SFTS were admitted to the ICU. During these clinical courses, disseminated intravascular coagulation (DIC) and encephalopathy occurred in two patients each. Of these patients, three (16%) died from disease progression.

### 3.2. Comparison of Standard Laboratory Parameters between SFTS and Rickettsiosis

Table S2 compares the standard laboratory parameters between JSF and ST in the rickettsiosis group. These laboratory findings of JSF and ST are similar, supporting the past surveillance data in Japan [20]. The hematological characteristics of patients with SFTS or rickettsiosis at the initial diagnosis are shown in Table 2. The levels of WBC, PLT, and CRP in the SFTS group were significantly lower than those in the rickettsiosis group (*p* < 0.001). Meanwhile, the levels of Hb, AST, ALT, and CK in the SFTS group were significantly higher than those in the rickettsiosis group (*p* < 0.01). Slightly significant differences in the T-Bil and PT-INR levels were found between the two groups (*p* < 0.05). The levels of Cr and BUN were not significantly different between the two groups.

### 3.3. Diagnostic Performance of Standard Laboratory Parameters

The ROC curve was used to identify the optimal diagnostic items for SFTS based on routine laboratory data, and the AUCs were calculated (Table 3). Among them, the WBC count and CRP levels were good markers for differentiating SFTS from rickettsiosis, and their AUCs were 0.97 [95% CI 0.93–1] and 0.98 [95% CI 0.93–1], respectively. Moreover, the AUCs used by PLT, AST, and LDH to predict the differentiation were 0.88 [95% CI 0.78–0.98], 0.83 [95% CI 0.72–0.95], and 0.81 [95% CI 0.68–0.93], respectively. The AUCs of other indicators (Hb, ALT, T-Bil, CK, Cr, and PT-INR) were <0.80.

### 3.4. Predictive Factors for Differentiating SFTS from Rickettsiosis

Table 4 shows the AUC, sensitivity, specificity, and positive and negative likelihood ratios of the clinical characteristics and standard laboratory parameters, which included the following nine factors: season group, absence of skin rash, absence of eschar, gastrointestinal symptoms, WBC, PLT, AST, LDH, and CRP. The AUC for the no skin rash factor was the highest at 0.98 [95% CI, 0.95–1]. Therefore, the presence of a skin rash can rule out SFTS (sensitivity, 95.6%; specificity, 100%). In addition, a low CRP level was a good laboratory parameter for discriminating patients with SFTS, and its cutoff point was 1.66 mg/dL (AUC, 0.97 [95% CI, 0.92–1]; sensitivity, 94.7%; specificity, 100%). Then, the presence of gastrointestinal symptoms had a high AUC (0.95, [95% CI, 0.88–1]) with a sensitivity of 89.5% and specificity of 100%. Similarly, leukopenia was also an excellent parameter, and the cutoff WBC count was 3.7 × 10^9^/L (AUC, 0.95 [95% CI, 0.88–1]; sensitivity, 89.5%; specificity, 100%). Meanwhile, thrombocytopenia was a good parameter, and the cutoff PLT count was 8.9 ×10^9^/L (AUC, 0.84 [95% CI, 0.76–0.94]; sensitivity, 89.5%; specificity, 80.0%). These findings suggest that the presence of gastrointestinal symptoms, absence of skin rash, leukopenia (<3.7 × 10^9^/L), and low CRP levels (<1.66 mg/dL) were superior as diagnostic prediction factors of SFTS compared to other factors.

**Figure 1:**
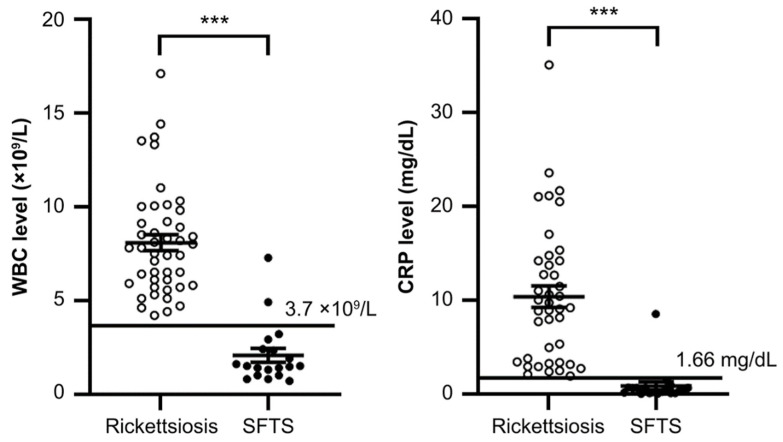
Comparison of white blood cell (WBC) counts and C-reactive protein (CRP) levels between rickettsiosis and severe fever with thrombocytopenia syndrome. The *p*-value of the Mann–Whitney test is shown in each graph. *** *p* < 0.0001.

Representative findings with regard to WBC and CRP levels are shown in Figure 1. Although there were significant differences in WBC and CRP levels between these two groups, a few patients with outlier counts in either WBC or CRP levels were observed.

## 4. Discussion

In the past, the Wakayama prefecture (particularly the southern region) had a high incidence of both JSF and ST [1,2,3]. In 2014, we first described a female patient with SFTS from this region, and the number of cases has increased annually [5,10,24]. Even now, Wakayama prefecture is a hotspot for emerging tick-borne infections with an increasing spread. These infections are considered a threat in the context of climate change [26]. In this study, SFTS cases were detected in all seasons, with peaks observed in summer. Moreover, JSF occurred from spring to autumn and peaked in summer. Notably, JSF and SFTS have similar seasonal characteristics. Meanwhile, in Wakayama prefecture, which is located south of 37° N, patients with ST are concentrated between October and December. These results are roughly consistent with recent reports from the National Surveillance and are attributed to the differences in pathogen-carrying mites or ticks and their activity [2,5]. These findings support the significance of the difference in onset season. Tick-borne infections were commonly observed among elderly people living in rural areas, consistent with previous reports [2,3,4,5,27]. No significant difference in comorbidities was found between SFTS and rickettsiosis. Most fatal cases of rickettsiosis and SFTS occurred in elderly people [2,4]. An older age was a critical risk factor for fatality due to SFTS [28,29]. For healthcare in these communities, tick-borne infections should be one of the differential diagnoses for febrile elderly outpatients.

Rickettsiosis including JSF and ST responds well to tetracyclines; however, treatment delay leads to severe disease and increases the risk of complications [7,30]. Therefore, early diagnosis, hospital admission, and rapid treatment are important for these patients. On the contrary, no antiviral drugs are currently effective for SFTS, and the optimal treatment strategy remains unknown [5,6]. Meanwhile, DIC, encephalopathy, and myocarditis, which exhibit rapid disease progression and high mortality, are among the complications of this infectious disease [11,12,13,21]. Thus, symptomatic and supportive therapy is crucial for the current treatment strategy of SFTS [5,6]. In addition, person-to-person transmission of SFTS has been reported through blood or bodily secretions, and nosocomial clusters sometimes occurred less frequently [16,17,18,19]. Accordingly, regardless of an early diagnosis, patients with SFTS should be considered for admission to high-volume centers with an ICU and infection control management. However, tick-borne infections are encountered frequently in the first presentation to primary care physicians as a fever of unknown origin. Furthermore, a definitive diagnosis requires PCR testing of blood samples or eschars, which often takes a few days [5,6,22,23]. These findings indicate the importance of early diagnosis of SFTS and rickettsiosis and differentiation between the two at the initial visit based on clinical characteristics and standard laboratory parameters. Some previous studies have already attempted to differentiate SFTS from JSF or ST using easy prediction tools [23,31,32,33,34].

Fever, eschar, and skin rashes are the main symptoms of rickettsiosis [23,31,32,33]. However, patients with rickettsiosis did not all manifest the triad and often exhibited nonspecific symptoms such as headaches and myalgia [23,31,32,33]. Patients with these symptoms might have been misdiagnosed and subsequently given inappropriate treatment [23,31,32,33,34,35]. A skin rash is rarely observed in patients with SFTS; however, the frequency was reported to range from 14% to 34.8% [23,31,32,33,34]. In this study, a skin rash was not observed in any of the patients with SFTS. Consistently, the AUC of no skin rash was the highest (0.98), being the most remarkable variable. Gastrointestinal symptoms (particularly diarrhea) are frequently found in patients with SFTS [23,31,32,33]. In this study, gastrointestinal symptoms were also present in 89% of the patients with SFTS. However, gastrointestinal symptoms occurred in 20–40% of patients with rickettsiosis [23,31,32,33]. These findings suggest that some patients with tick-borne infections have overlapping characteristics (skin rashes and gastrointestinal symptoms), which makes differentiating tick-borne infections by these symptoms challenging.

In clinics and hospitals, the WBC count and CRP level should be urgently obtained using laboratory testing. Some reports have indicated that leukopenia (<4.0 × 10^9^/L) and a low CRP level (<1.0 mg/dL) were useful in differentiating SFTS from rickettsiosis [23,31,32,33,34]. Notably, a low CRP level was the most useful for diagnostic differentiation [23,31,32]. Our results also showed that leukopenia (<3.7 × 10^9^/L) and a low CRP level (<1.66 mg/dL) were significant predictive parameters in differentiating SFTS from rickettsiosis, with AUC levels of 0.95 and 0.97, respectively. In contrast, we also observed an SFTS patient with both leukocytosis and a high CRP level. Consequently, this patient with SFTS also had *Escherichia coli* bacteremia. Among standard laboratory parameters, LDH and AST levels were significantly high in the SFTS group but were not found to be predictive parameters by statistical analysis. Although two previous reports have shown that prolonged activated partial thromboplastin was also helpful for differential diagnosis [31,34], our analysis was insufficient because of missing values. Therefore, if patients with clinically suspected tick-borne infections exhibit leukopenia and low CRP levels, primary care physicians should consider SFTS and transfer them to a hospital that provides intensive care. Moreover, standard and, if needed, greater precautions are recommended for these physicians when examining patients who are suspected to have tick-borne infections, particularly SFTS [36].

This study has some limitations. First, the study was retrospectively conducted by collecting data on patients from three hospitals with ICUs and one without an ICU. A selection bias toward the treatment strategy was probable. Second, for all patients, the number of laboratory parameters was small, and not all tests, including CRP and PT-INR, were performed. Third, a few facilities in Wakayama prefecture did not participate in the study; therefore, the number of SFTS cases was small. Hence, to evaluate their value in real-world practice, a prospective study using the variables identified in this study to differentiate SFTS from rickettsiosis with more patients is necessary.

## 5. Conclusions

In this study, we suggested a diagnostic prediction tool for SFTS in comparison with rickettsiosis, which consists of four variables: the presence of gastrointestinal symptoms, the absence of skin rash, leukopenia (<3.7 × 10^9^/L), and low CRP levels (<1.66 mg/dL). In the co-endemic Wakayama prefecture, these variables might be useful at the first hospital visit as a diagnostic support tool for patients with SFTS, even in rural communities. This approach might be a strategy for improving the outcomes of life-threatening tick-borne infections in Japan, which requires real-time information sharing.

## Figures and Tables

**Table 1:** Patient clinical characteristics at diagnosis between rickettsiosis and severe fever with thrombocytopenia syndrome.

Characteristics	Total (*n* = 64)	Rickettsiosis (*n* = 45)	SFTS (*n* = 19)	*p*-Value
age (years)				0.14
median [IQR]	75.5 [68.5–84]	79 [69–85]	74 [63–81]	
sex, *n* (%)				0.83
male	29 (45)	20 (44)	9 (47)	
female	35 (51)	25 (56)	10 (53)	
comorbidities, *n* (%)				
hypertension	25 (39)	15 (33)	10 (53)	0.15
diabetes mellitus	8 (12)	4 (9)	4 (21)	0.18
hepatic disease	1 (2)	1 (2)	0 (0)	0.51
chronic kidney disease	0 (0)	0 (0)	0 (0)	
cardiac disease	12 (19)	11 (24)	1 (5)	0.072
pulmonary disease	2 (3)	1 (2)	1 (5)	0.52
cancer	7 (11)	5 (11)	2 (11)	0.95
season, *n* (%)				0.26
spring	7 (11)	4 (9)	3 (16)	
summer	28 (44)	17 (38)	11 (58)	
autumn	19 (30)	16 (36)	3 (16)	
winter	10 (16)	8 (18)	2 (11)	
season group				0.047
spring–summer	35 (55)	21 (47)	14 (74)	
autumn–winter	29 (45)	24 (53)	5 (26)	
eschar, *n* (%)				0.046
presence	42 (66)	33 (73)	9 (47)	
skin rash, *n* (%)				<0.001
presence	43 (67)	43 (96)	0 (0)	
gastrointestinal symptoms, *n* (%)				<0.001
vomiting	4 (6)	0 (0)	4 (21)	
abdominal pain	9 (14)	0 (0)	9 (47)	
diarrhea	9 (14)	0 (0)	9 (47)	
>2 symptoms	17 (27)	0 (0)	17 (89)	
treatments, *n* (%)				
antimicrobial	57 (89)	45 (100)	12 (63)	
steroid	2 (3)	0 (0)	2 (11)	
immunoglobulin replacement	2 (3)	0 (0)	2 (11)	
outcome, *n* (%)				
ICU admission	9 (14)	1 (2)	8 (42)	
death	4 (6)	1 (2)	3 (16)	

ICU, intensive care unit; IQR, interquartile range; SFTS, severe fever with thrombocytopenia syndrome.

**Table 2:** Clinical laboratory findings at diagnosis between rickettsiosis and severe fever with thrombocytopenia syndrome.

Variable	Total (*n* = 64)	Rickettsiosis (*n* = 45)	SFTS (*n* = 19)	*p*-Value
WBC (×10^9^/L)	*n* = 64	*n* = 45	*n* = 19	<0.001
median [IQR]	6.25 [3.06–8.45]	7.8 [5.90–9.20]	1.5 [1.00–2.41]	
Hb (g/dL)	*n* = 64	*n* = 45	*n* = 19	0.006
median [IQR]	13.6 [12.1–15.0]	13.3 [12.0–14.5]	15.1 [13.4–15.8]	
PLT (×10^9^/L)	*n* = 64	*n* = 45	*n* = 19	<0.001
median [IQR]	10.55 [7.40–13.35]	11.8 [9.60–16.1]	5.10 [3.30–8.30]	
AST (IU/L)	*n* = 64	*n* = 45	*n* = 19	<0.001
median [IQR]	63 [44–130]	53 [39–79]	161 [91–390]	
ALT (IU/L)	*n* = 64	*n* = 45	*n* = 19	0.001
median [IQR]	47.5 [29–90.5]	42 [25–61]	83 [48–135]	
T-Bil (IU/L)	*n* = 63	*n* = 44	*n* = 19	0.028
median [IQR]	0.6 [0.5–0.8]	0.6 [0.5–0.9]	0.5 [0.4–0.6]	
LDH (IU/L)	*n* = 63	*n* = 44	*n* = 19	<0.001
median [IQR]	384 [280–541]	353 [266–421.5]	559 [384–1039]	
CK (mg/dL)	*n* = 62	*n* = 43	*n* = 19	0.002
median [IQR]	166 [102–398]	135 [78–235]	308 [155–2127]	
Cr (mg/dL)	*n* = 64	*n* = 45	*n* = 19	0.25
median [IQR]	1.0 [0.7–1.2]	0.9 [0.8–1.2]	1.1 [0.7–1.5]	
BUN (mg/dL)	*n* = 63	*n* = 44	*n* = 19	0.085
median [IQR]	19.5 [14–27]	19.1 [14–24.1]	25.3 [15–38]	
CRP (mg/dL)	*n* = 60	*n* = 41	*n* = 19	<0.001
median [IQR]	4.4 [0.7–11.2]	9.2 [3.4–14.2]	0.4 [0.1–0.7]	
PT-INR	*n* = 52	*n* = 33	*n* = 19	0.017
median [IQR]	1.1 [1.0–1.2]	1.1 [1.1–1.2]	1.0 [1.0–1.1]	

ALT, alanine aminotransferase; AST, aspartate aminotransferase; BUN, blood urea nitrogen; CK, creatine kinase; Cr, creatinine; CRP, C-reactive protein; Hb, hemoglobin; IQR, interquartile range; LDH, lactate dehydrogenase; PLT, platelet; PT-INR, prothrombin time-international normalized ratio; SFTS, severe fever with thrombocytopenia syndrome; T-Bil, total bilirubin; WBC, white blood cell.

**Table 3:** Areas under the curve of laboratory findings at diagnosis.

	*n*	AUC	SE	95% CI
WBC	64	0.97	0.023	0.93–1
Hb	64	0.72	0.081	0.56–0.88
PLT	64	0.88	0.049	0.78–0.98
AST	64	0.83	0.059	0.72–0.95
ALT	64	0.75	0.064	0.63–0.88
T-Bil	63	0.67	0.074	0.53–0.82
LDH	63	0.81	0.064	0.68–0.93
CK	62	0.75	0.066	0.62–0.88
Cr	64	0.59	0.086	0.42–0.76
BUN	63	0.64	0.087	0.47–0.81
CRP	60	0.98	0.022	0.93–1
PT-INR	52	0.70	0.082	0.54–0.86

AUC, area under the curve; CI, confidence interval; SE, standard error.

**Table 4:** Predictive accuracy of each variable on differentiating SFTS from rickettsiosis.

	*n*	AUC	95% CI	Sensitivity	Specificity	LR+	LR−	*p*-Value
spring–summer	64	0.63	0.51–0.76	53.3%	73.7%	2.03	0.63	0.047
absence of skin rash	64	0.98	0.95–1	95.6%	100%	−	0.04	<0.001
absence of eschar	64	0.63	0.50–0.76	73.3%	52.6%	1.55	0.51	0.046
presence of gastrointestinal symptoms	64	0.95	0.88–1	89.5%	100%	−	0.11	<0.001
WBC < 3.7 × 10^9^/L	64	0.95	0.88–1	89.5%	100%	−	0.11	<0.001
PLT < 8.9 × 10^9^/L	64	0.84	0.76–0.94	89.5%	80.0%	4.45	0.13	<0.001
AST > 85.5 IU/L	64	0.79	0.65–0.89	79.0%	80.0%	3.95	0.26	<0.001
LDH > 500 IU/L	63	0.75	0.63–0.87	68.4%	82.2%	3.85	0.38	<0.001
CRP < 1.66 mg/dL	60	0.97	0.92–1	94.7%	100%	−	0.05	<0.001

LR+, positive likelihood ratios; LR−, negative likelihood ratios.

## Data Availability

The data presented in this study are available on request from the corresponding author.

## Supplementary Materials

The following supporting information can be downloaded at: https://www.mdpi.com/article/10.3390/medicina59112024/s1, Table S1: Patient clinical characteristics at diagnosis between Japanese spotted fever and scrub typhus; Table S2: Clinical laboratory findings at diagnosis between Japanese spotted fever and scrub typhus.
