# Supplementary material for: Clinical Characteristics and Diagnostic Prediction of Severe Fever with Thrombocytopenia Syndrome and Rickettsiosis in the Co-Endemic Wakayama Prefecture, Japan
Source: Medicina (Kaunas). 2023 Nov 17;59(11):2024. doi: 10.3390/medicina59112024 (PMC10672843; doi:10.3390/medicina59112024)
Supplement: Supplementary file 1 [file medicina-59-02024-s001.zip › medicina-2649637-supplementary.pdf]

Supplementary Material

Table S1. Patient clinical characteristics at diagnosis between Japanese spotted fever and scrub typhus.

Characteristics	Total (*n* = 45)	JSF (*n* = 26)	ST (*n* = 19)	*p*-value
age (years)				0.43
median [IQR]	79 [69–85]	80 [74–85]	75 [65–85]	
sex, *n* (%)				0.03
male	20 (44)	8 (31)	12 (63)	
female	25 (56)	18 (69)	7 (37)	
comorbidities, *n* (%)				
hypertension	15 (33)	8 (31)	7 (37)	0.67
diabetes mellitus	4 (9)	2 (8)	2 (11)	0.74
hepatic disease	1 (2)	0 (0)	1 (5)	0.24
chronic kidney disease	0 (0)	0 (0)	0 (0)	
cardiac disease	11 (24)	6 (23)	5 (26)	0.8
pulmonary disease	1 (2)	1 (4)	0 (0)	0.39
cancer	5 (11)	1 (4)	4 (21)	0.07
season, *n* (%)				<0.001
spring	4 (9)	4 (15)	0 (0)	
summer	17 (38)	17 (65)	0 (0)	
autumn	16 (36)	5 (19)	11 (58)	
winter	8 (18)	0 (0)	8 (42)	
season group				<0.001
spring–summer	21 (47)	21 (81)	0 (0)	
autumn–winter	24 (53)	5 (19)	19 (100)	
eschar, *n* (%)				0.036
presence	33 (73)	16 (62)	17 (89)	
skin rash, *n* (%)				0.22
presence	43 (96)	24 (92)	19 (100)	
gastrointestinal symptoms, *n* (%)				
vomiting	0 (0)	0 (0)	0 (0)	
abdominal pain	0 (0)	0 (0)	0 (0)	
diarrhea	0 (0)	0 (0)	0 (0)	
>2 symptoms	0 (0)	0 (0)	0 (0)	

IQR, interquartile range; JSF, Japanese spotted fever; ST, scrub typhus

Table S2. Clinical laboratory findings at diagnosis between Japanese spotted fever and scrub typhus.

**Variable**	**Total (*n* = 45)**	**JSF (*n* = 26)**	**ST (*n* = 19)**	**p-value**
WBC (×10⁹/L)	*n* = 45	*n* = 26	*n* = 19	0.073
median [IQR]	7.8 [5.90–9.20]	8.15 [6.50–10.04]	6.5 [5.3–8.4]	
Hb (g/dL)	*n* = 45	*n* = 26	*n* = 19	0.051
median [IQR]	13.3 [12.0–14.5]	12.6 [11.4–14.2]	13.6 [12.7–14.8]	
PLT (x10⁹/L)	*n* = 45	*n* = 26	*n* = 19	0.047
median [IQR]	11.8 [9.60–16.1]	11.25 [8.10–13.3]	13.1 [10.5–18.2]	
AST (IU/L)	*n* = 45	*n* = 26	*n* = 19	0.73
median [IQR]	53 [39–79]	53.5 [35–108]	53 [43–62]	
ALT (IU/L)	*n* = 45	*n* = 26	*n* = 19	0.58
median [IQR]	42 [25–61]	42 [27–74]	42 [23–53]	
T-Bil (IU/L)	*n* = 44	*n* = 26	*n* = 18	0.023
median [IQR]	0.6 [0.5–0.9]	0.6 [0.5–0.7]	0.9 [0.6–1.2]	
LDH (IU/L)	*n* = 44	*n* = 26	*n* = 18	0.28
median [IQR]	353 [266–421.5]	315 [257–432]	371 [314–418]	
CK (mg/dL)	*n* = 43	*n* = 26	*n* = 17	0.087
median [IQR]	135 [78–235]	144.5 [107–353]	102 [64–222]	
Cr (mg/dL)	*n* = 45	*n* = 26	*n* = 19	0.6
median [IQR]	0.9 [0.8–1.2]	0.9 [0.7–1.2]	0.8 [0.8–1.1]	
BUN (mg/dL)	*n* = 44	*n* = 26	*n* = 18	0.53
median [IQR]	19.1 [14–24.1]	19.5 [14–26.9]	18.7 [15.5–22.9]	
CRP (mg/dL)	*n* = 41	*n* = 22	*n* = 19	0.053
median [IQR]	9.2 [3.4–14.2]	10.5 [4.9–20.5]	8.1 [2.9–12.7]	
PT-INR	*n* = 33	*n* = 18	*n* = 15	0.25
median [IQR]	1.1 [1.1–1.2]	1.1 [1.1–1.1]	1.2 [1.1–1.3]	

ALT, alanine aminotransferase; AST, aspartate aminotransferase; BUN, blood urea nitrogen; CK, creatine kinase; Cr, creatinine; CRP, C-reactive protein; Hb, hemoglobin; IQR, interquartile range; JSF, Japanese spotted fever; LDH, lactate dehydrogenase; PLT, platelet; PT-INR, prothrombin time-international normalized ratio; ST, scrub typhus; T-Bil, total bilirubin; WBC, white blood cell
